# *BTK* mutations selectively regulate BTK expression and upregulate monocyte *XBP1* mRNA in XLA patients

**DOI:** 10.1002/iid3.57

**Published:** 2015-06-04

**Authors:** Marcelo A Teocchi, Vanessa Domingues Ramalho, Beatriz M Abramczuk, Lília D'Souza-Li, Maria Marluce Santos Vilela

**Affiliations:** 1 Center for Investigation in Pediatrics, University of Campinas (UNICAMP)Campinas, São Paulo, Brazil; 2 Department of Pediatrics, Faculty of Medical Sciences, University of Campinas (UNICAMP)Campinas, São Paulo, Brazil

**Keywords:** Endoplasmic reticulum stress, HSP90B1, interleukin 6, unfolded protein response (UPR), X-linked agammaglobulinemia

## Abstract

Mutations in the Bruton agammaglobulinemia tyrosine kinase (*BTK*) gene are responsible for X-linked agammaglobulinemia (XLA). Unfolded or misfolded proteins can trigger stress pathways in the endoplasmic reticulum (ER), known as unfolded protein response (UPR). The aim was to clarify the involvement of UPR in XLA pathophysiology. By reverse transcription-quantitative PCR, we evaluated the expression of *BTK* and 12 UPR-related genes in eight patients. Moreover, we assessed the BTK protein expression and pattern in the patients' monocytes by flow cytometry and fluorescence immunocytochemistry. We found a reduced *BTK* expression in patients with stop codon mutations (*P* < 0.02). However, missense mutations did not affect *BTK* expression. Flow cytometry showed a reduction of BTK in patients which was corroborated by an absent or nonfunctional protein synthesis revealed by immunocytochemistry. In contrast with the other UPR-related genes, X-box binding protein 1 (*XBP1*) was markedly upregulated in the patients (*P* < 0.01), suggesting Toll-like receptor (TLR) activation since BTK directly interacts with TLRs as a negative regulator and XBP1 can be activated in direct response to TLR ligation. Different *BTK* mutations can be identified by the *BTK* expression. Inasmuch as UPR-related genes were downregulated or unaltered in patients, we speculate the involvement of the TLRs-XBP1 axis in the XLA pathophysiology. Such data could be the basis for further studies of this novel pathomechanism concerning XLA.

## Introduction

X-linked agammaglobulinemia (XLA, OMIM # 300755) is characterized by an end in differentiation of B lymphocytes in bone marrow, leading to profound hypogammaglobulinemia, few or absent peripheral B lymphocytes, and recurrent infections with encapsulated bacteria and enteroviruses 1,2. Bruton agammaglobulinemia tyrosine kinase (*BTK*) is the gene which causes XLA when functionally mutated 3,4. It encompasses 37.5 kb containing 19 exons and encodes a multidomain protein composed of five different domains 4,5.

Mutations have been found in all *BTK* domains, being spread throughout the gene (http://bioinf.uta.fi/BTKbase) and have been reported in many different countries 6. Our group has been the first to publish the diagnosis of XLA by analysis of mutations of the *BTK* gene in Brazilian patients 7,8. Despite the large number of mutations identified, it has not been possible to correlate the genetic defect with the severity of the resulting clinical phenotype 9. Expression analyses in XLA patients have shown that most of these individuals do not express BTK protein regardless of the mutations they have 10–13. However, it is not clear how specific mutations may affect the function of BTK and cause XLA.

Unfolded or misfolded proteins can trigger stress pathways in the endoplasmic reticulum (ER), called unfolded protein response (UPR). Stress pathways of the ER have been implicated in inflammatory diseases 14 but not yet studied in XLA patients. There are three ER stress branches of sensors: ERN1 (endoplasmic reticulum to nucleus signaling 1) (also known as IRE1 [inositol-requiring protein 1]), EIF2AK3 (eukaryotic translation initiation factor 2-alpha kinase 3) (also known as PERK [PKR-like-ER kinase]), and ATF6 (activating transcription factor 6). The ERN1-XBP1(X-box binding protein 1) pathway is the most highly conserved and is critical for ER biogenesis and the secretory capacity of cells 15.

The amount of *XBP1* mRNA is critical to the production of the active (spliced) form of (s)XBP1 16; the *XBP1* mRNA level is kept low in non-stressed cells and its transcription is induced under ER stress 17. As UPR is constitutively active at a basal level 18–20, regulation through induction or repression of *XBP* expression allows the maintenance of ER homeostasis 15. Moreover, *XBP1* activity is regulated at multiple levels and may modulate ER homeostasis independently of classic UPR activation 15. Studies demonstrated that *XBP1* plays a role in innate and adaptive immune responses such as in the terminal differentiation of B lymphocytes to plasma cells 21, effector CD8^+^ T cells 22, in dendritic cell survival 23, immunoglobulin secretion 24, and macrophage responses induced via Toll-like receptors 25.

In this study, we investigated the relation between the mutated *BTK* and UPR and report for the first time the overexpression of *XBP1* in monocytes from XLA patients. As an important multifunctional transcription factor that controls the expression of critical genes associated with the proper functioning of the immune system and the cellular stress response, the involvement of XBP1 on the XLA pathophysiology is conceivable.

## Methods

### Subjects

X-linked agammaglobulinemia was diagnosed according to the criteria of the World Health Organization scientific group for primary immunodeficiency diseases 26: low levels of circulating B cells (measured by levels of CD19-positive cells in blood samples), decreased or absent immunoglobulins in serum, and a typical clinical history with recurrent bacterial infection or a positive family history.

Eight male patients with X-linked agammaglobulinemia (median age 17.73; range from 6.36 to 32.02 years old) and eight healthy male volunteers (median age 18.62; range from 6.39 to 32.45 years old) were enrolled for the study. Patients were in a clinically stable situation without fever and not hospitalized. They were under intravenous human immunoglobulin therapy monthly and had no infectious intercurrences.

Monocyte UPR-related gene expression evaluations were carried out only in six patients (median age 14.36; range from 6.36 to 32.02) because two could not provide an adequate amount of cells. For statistical comparisons, the control group also consisted of six age- and gender-matched healthy volunteers.

This study was approved by the Research Ethics Committee of the University of Campinas (UNICAMP), Campinas, São Paulo, Brazil (#759/2005). Clinical characteristics, including levels of immunoglobulins and B cell number, are described in Table1.

**Table 1:** Clinical and laboratorial characteristics of X-linked agammaglobulinemia patients

Patient	Age (years)	Age at onset	Age at diagnosis^a^	Family history^b^	Ig levels at diagnosis (mg/dL)			CD19^+^ (%)	BTK expression^c^	Mutations		Domain
					IgG	IgM	IgA			Nucleotide	Protein	
XLA01	20.67	4 y	6 y	+	298 (750–1780)	6 (28–212)	20 (90–450)	0.04	26.30%	215G>T	R28L	PH
XLA02	24.11	6 y	6 y	+	180 (750–1780)	18 (28–212)	22 (90–450)	0.30	25.60%	1970G>A	G613D	SH1
XLA03	24.76	6 y	6 y	+	UD (750–1780)	UD (28–212)	3 (90–450)	0.09	9.00%	251A>G	Y40C	PH
XLA04	6.36	No infection	11 mo	+	126 (282–1115)	31 (40–156)	18 (12–104)	0.14	not tested	718C>T	Q196X	TH
XLA05	9.31	3 mo	2y8mo	+	149 (610–1610)	22 (29–195)	1 (40–289)	0.30	39.00%	63104delT	Glu348fsX55	SH2
XLA06	13.94	4 mo	2 y	+	86 (610–1610)	26 (29–195)	23 (40–289)	0.10	4.52%	62854_62855insT	Thr324fsX25	SH2
XLA07	14.80	No infection	1 y 6 mo	+	Unregistered	Unregistered	Unregistered	0.09	3.00%	IVS5+1G>A	Exon 5 skipping	PH
XLA08	32.02	2 y	8 y	−	54 (750–1780)	UD (28–212)	UD (90–450)	0.10	13.30%	1634T>C	M501T	SH1

UD, undetectable.

a Age at the start of intravenous immunoglobulin replacement.

b “+” indicates that boy(s) in the same family died at a young age because of infection.

c cNormal expression is >95%.

### Detection of *BTK* mutations

Briefly, peripheral blood mononuclear cells (PBMC) were prepared from venous blood using Ficoll-Hypaque separation. Total RNA was extracted from PBMC with TRIzol® Reagent (Life Technologies, Carlsbad, CA) and used for first-strand cDNA synthesis. Bruton agammaglobulinemia tyrosine kinase PCR amplifications involved seven overlapping primers 10. To confirm the detected mutation, genomic DNA was purified from venous blood with a Gentra Puregene Blood Kit (QIAGEN, Life Technologies) and amplified with primers encompassing the changed region in the *BTK* gene 27. Amplicons were sequenced by an ABI 3730 DNA Analyzer (Life Technologies).

### Reverse transcription-quantitative PCR (RT-qPCR)

To extract total RNA, 1 mL of TRIzol® Reagent (Life Technologies) was added per 5–10 × 10^6^ of PBMC samples, homogenized, and then further processed according to the manufacturer's instructions. Subsequently, 1 µg of total RNA of each sample was reverse transcribed into cDNA using 200 U of Superscript III Reverse Transcriptase (Life Technologies) and 3 µg of Random Primers (Life Technologies) again according to the manufacturer's instructions. To assess the expression of ER stress sensors on monocytes, CD14^+^ monocyte subpopulation was isolated from PBMC using a magnetic cell sorting system in accordance with the manufacturer's protocol (Miltenyi Biotec, Bergisch Gladbach, Germany). Total RNA was extracted from monocytes with an illustra RNAspin Mini Isolation kit (GE Healthcare, Buckinghamshire, UK) and similarly reverse transcribed into cDNA.

Complementary DNA samples derived from the investigated genes (Table2) were detected using an ABI PRISM 7500 Sequence Detection System (Life Technologies) and TaqMan Gene Expression Assays (Life Technologies): 5′-FAM-labeled probes and corresponding primer pairs.

**Table 2:** List of reference and target genes

Approved symbol	Task	Assay ID	Approved name	Synonyms
ATF4	UPR-related target	Hs00909569_g1	Activating transcription factor 4	CREB-2, “tax-responsive enhancer element B67“, TAXREB67
ATF6	UPR-related target	Hs00232586_m1	Activating transcription factor 6	“Activating transcription factor 6 alpha,“ ATF6A
BAX	UPR-related target	Hs00180269_m1	BCL2-associated X protein	BCL2L4
BCL2	UPR-related target	Hs00608023_m1	B-cell CLL/lymphoma 2	Bcl-2, PPP1R50, “protein phosphatase 1, regulatory subunit 50“
BTK	XLA target	Hs00163761_m1	Bruton agammaglobulinemia tyrosine kinase	ATK, PSCTK1, XLA
CALR	UPR-related target	Hs00189032_m1	Calreticulin	“Autoantigen Ro,“ cC1qR, CRT, FLJ26680, RO, “Sicca syndrome antigen A (autoantigen Ro; calreticulin),“ SSA
DDIT3	UPR-related target	Hs01090850_m1	DNA-damage-inducible transcript 3	“C/EBP zeta,“ CHOP, CHOP10, GADD153
EIF2AK3	UPR-related target	Hs00984006_m1	Eukaryotic translation initiation factor 2-alpha kinase 3	PEK, PERK
EIF2S1	UPR-related target	Hs00187953_m1	Eukaryotic translation initiation factor 2, subunit 1 alpha, 35 kDa	EIF-2alpha, EIF2A
ERN1	UPR-related target	Hs00176385_m1	Endoplasmic reticulum to nucleus signaling 1	“Inositol-requiring enzyme 1,“ IRE1, IRE1P
GAPDH	Reference gene	Hs99999905_m1	Glyceraldehyde-3-phosphate dehydrogenase	
HPRT1	Reference gene	Hs99999909_m1	Hypoxanthine phosphoribosyltransferase 1	HGPRT, “Lesch-Nyhan syndrome“
HSPA5	UPR-related target	Hs99999174_m1	Heat shock 70kDa protein 5 (glucose-regulated protein, 78 kDa)	BiP
HSP90B1	UPR-related target	Hs00427665_g1	Heat shock protein 90 kDa beta (Grp94), member 1	GP96, GRP94
IL6	Interleukin	Hs00985639_m1	Interleukin 6	BSF2, HGF, HSF, IL-6, “interferon, beta 2“
XBP1	UPR-related target	Hs00231936_m1	X-box binding protein 1	

Gene names are in accordance with the approved symbol from the HUGO Gene Nomenclature Committee (HGNC) database.

Each qPCR was run as triplicates with a cDNA sample of 10 ng in 6.25 µL of TaqMan Gene Expression Master Mix (Life Technologies), 0.625 µL of the respective probe/primer mix, and 0.625 µL of purified and deionized H_2_O. Relative gene expression quantification data were generated and analyzed using the 7500 Software version 2.0.5 (Life Technologies). Expression levels of genes were calculated with the 2^−ΔΔCt^ method using the combination of *GAPDH* and *HPRT1* as the reference gene.

### Flow cytometry analysis of BTK expression

BTK protein quantification was calculated by flow cytometry 8,28. PBMC were removed from whole blood using Ficoll-Hypaque and later were stained with a phycoerythrin-labeled anti-CD14 (IgG2a; Dako, Japan) monoclonal antibody to separate monocytes. These cells were fixed in 4% paraformaldehyde in phosphate-buffered saline for 15 min at room temperature, permeabilized in 0.1% Triton X-100 for 5 min, incubated with 2 µg/mL anti-BTK (48-2H) or control IgG1 (Dako) monoclonal antibodies for 20 min on ice. Following this, they were washed and incubated again with a 1:1000 dilution of fluorescein isothiocyanate-conjugated goat antimouse IgG1 antibody (SouthernBiotech, Birmingham, AL) for 20 min on ice. The stained cells were analyzed by flow cytometry (Epics XL-MCL flow cytometer, Beckman Coulter, Pasadena, CA).

### Immunocytochemistry and confocal fluorescence microscopy

Peripheral blood mononuclear cells were pipetted onto a glass slide previously treated with acetic acid 2 N and 70% ethanol. Adherent cells were fixed in 4% paraformaldehyde in PBS and 0.12 M sucrose for 20 min at room temperature. For the process of permeabilization, cells were treated with 0.2% Triton X-100 in PBS for 20 min.

Non-specific binding sites were blocked using 6% goat serum. Cells were incubated overnight at 4°C with monoclonal anti-BTK (Santa Cruz Biotechnology, Inc., Santa Cruz, CA), that recognizes the epitope at amino acid positions 459-659 of the human BTK. Further, we used a FITC-conjugated goat anti-mouse IgG1 as the secondary antibody (SouthernBiotech – 1:1000). After extensive washing with PBS, the coverslips were incubated with Hoechst (Life Technologies) for visualization of the nucleus and mounted on slides using Prolong Gold antifade (Life Technologies). Images were generated in a confocal fluorescence microscope (LSM - 510 Meta, ZEISS, Jena, Germany) with a magnification of 63×.

### Statistical analysis

The GraphPad Prism 5 software for Windows (version 5.04) was used for statistical analyses (San Diego, CA; www.graphpad.com). Kruskal–Wallis and Dunn's Multiple Comparison tests were used to evaluate differences in the *BTK* expression among three groups: XLA patients with mutations that lead to a premature stop codon, XLA patients with missense mutations, and healthy controls. Regarding the expression of UPR-related genes, Mann–Whitney U test was applied to assess significant differences between XLA patients and controls. Statistical significance was determined as *P* < 0.05.

## Results

### Stop codon or missense mutations were identified in the XLA patients

Table1 shows the nucleotide change which occurred in each XLA patient and its consequences in the synthesis of the protein.

Four patients had missense mutations, where the substitution of one nucleotide (XLA01 215G>T, XLA02 1970G>A, XLA03 251A>G, and XLA08 1634T>C) leads to an aminoacid replacement in the protein, without the creation of a premature stop codon. However, these missense mutations affect known and conserved residues in *BTK*, with R28 being one of the most affected. In the PH domain, this residue is essential for binding to phosphatidyl inositol, allowing the protein to reach the plasma membrane 29. Furthermore, in the PH domain, the substitution of residue Y40 has implications for the conformation of the loop between β strands and the binding site with phosphatidyl inositol 30. The residue G613 is part of a structural assembly of functionally important residues of the BTK kinase domain 31. The G613D mutation may prevent the interaction of this domain with other BTK domains or with their substrates. The M501T mutation also affects the kinase domain, suggesting an interference with the enzymatic activity of BTK.

In four patients, XLA04 to XLA07, we identified premature terminations caused by nonsense mutation (718C>T), frameshifts (63104delT and 62854_62855insT), and a splice donor-site defect (IVS5+1G>A).

### Different *BTK* mutations lead to a specific expression profile for *BTK*

**Figure 1:**
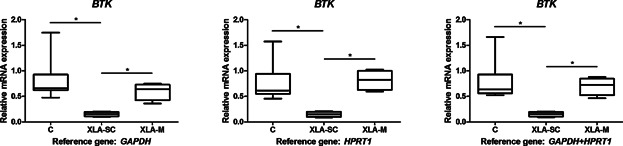
Expression of *BTK* in PBMC from XLA patients and healthy controls. Relative expression of the *BTK* gene in XLA patients with mutations that lead to a premature stop codon (XLA-SC; *n* = 4), XLA patients with missense mutations (XLA-M; *n* = 4), and controls (C; *n* = 8). The *y*-axis represents the quantitative data of the relative mRNA expression of the *BTK* gene. Data from RT-qPCRs were generated from three reference genes: *GAPDH*, *HPRT1*, and *GAPDH+HPRT1* (in combination). Data are shown as median with interquartile range (whiskers: minimum to maximum). Statistical analysis of the variance between the different groups was performed using the test of Kruskal–Wallis and Dunn's Multiple Comparison test was used to compare all pairs of data (*P < 0.05).

A distinct pattern of *BTK* expression was observed in patients with a premature stop codon and those with missense mutations (Fig. 1). Specifically, patients with mutations resulting in a premature stop codon exhibit reduced expression of the *BTK* gene (*P* < 0.02, regardless of the reference gene). However, missense mutations do not affect *BTK* expression.

### Absent or abnormal subcellular localization of BTK protein in the cell cytoplasm

**Figure 2:**
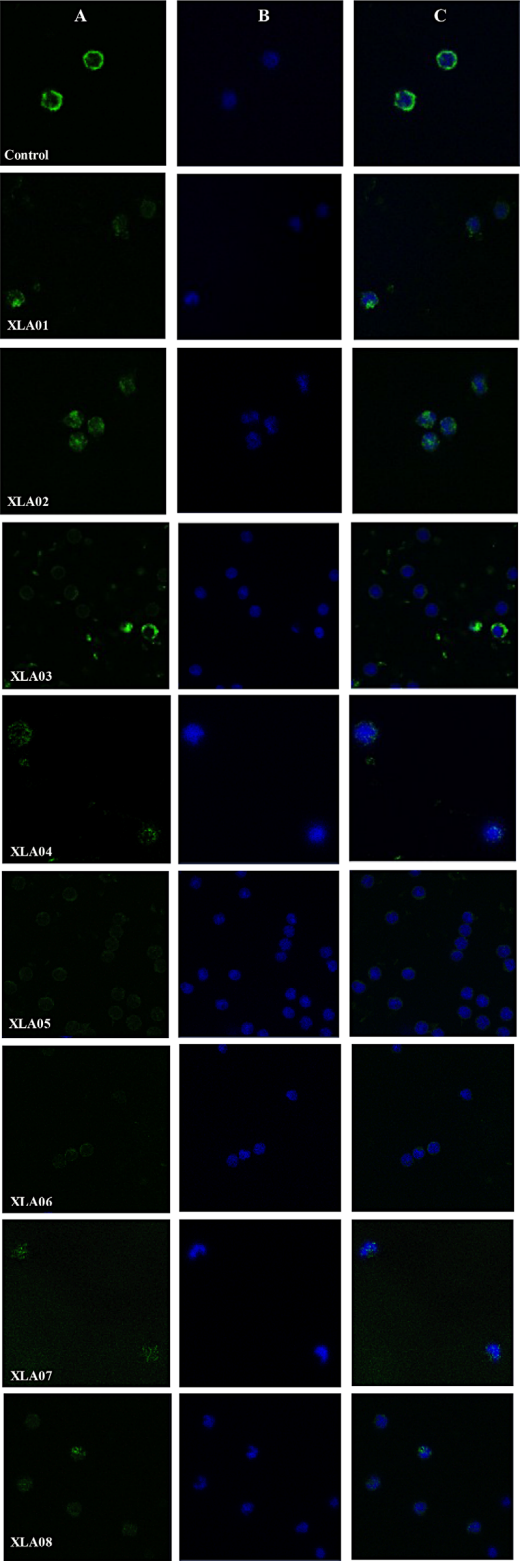
Immunolabeling of the BTK protein in PBMC (adherent monocytes) from XLA patients and healthy controls. Immunocytochemistry in PBMC from XLA patients with fluorescent labeling of BTK in green (A, FITC), the cell nucleus in blue (B, Hoechst), and merged in C. Mutations in *BTK* lead to a lack of BTK protein or nonfunctional protein synthesis, evidenced by abnormal subcellular localization of BTK protein in the cell cytoplasm. Patients XLA01, XLA02, XLA03, and XLA08 have missense mutations. Patients XLA04, XLA05, XLA06, and XLA07 have premature terminations caused by nonsense mutation. Original magnification of 63x.

The monocyte BTK expression evaluated by flow cytometry revealed a BTK deficiency (3.0–39.0%) in the seven patients analyzed (Table1). By immunocytochemistry, we found that mutations in *BTK* lead to a lack of BTK protein or nonfunctional protein synthesis, evidenced by abnormal subcellular localization of BTK protein in the cell cytoplasm (Fig. 2). The absence of functional BTK is further confirmed by the very low levels of circulating B lymphocytes observed in all patients studied (CD19^+^ percentage; Table1).

### Increased expression of XBP1 in monocytes from XLA patients

**Figure 3:**
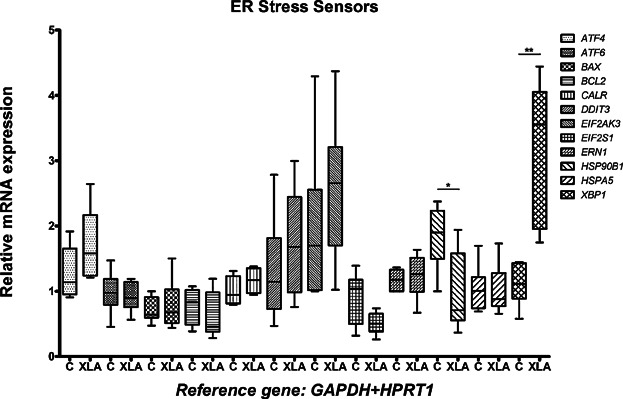
Expression of ER stress sensor genes in monocytes from XLA patients and healthy controls. Relative expression of 12 genes associated with UPR and the ER stress (see legend and Table2) in XLA patients (XLA; *n* = 6) and healthy controls (C; *n* = 6). The *y*-axis represents the quantitative data of the relative mRNA expression of genes. Data from RT-qPCRs were generated from *GAPDH+HPRT1* (in combination) as the reference gene. Data are shown as median with interquartile range (whiskers: minimum to maximum). Statistical analyses of the variance between the two groups (C vs. XLA) were performed using Mann–Whitney test (*P < 0.05; **P < 0.01).

To elucidate the molecular features of UPR on XLA patients, RT-qPCR analysis was performed on CD14^+^ cells isolated from six patients with XLA and six healthy volunteers. Among the 12 evaluated genes, the expression of *XBP1* was significantly higher (*P* = 0.0022) in XLA patients than in healthy volunteers (Fig. 3). On the other hand, *HSP90B1* expression was lower in XLA patients (*P* = 0.026).

Additionally, we also quantified *IL6* expression. It is known that sXBP1 induces IL6 secretion. We found an *IL6* upregulation (*P* < 0.02, *GAPDH+HPRT1* as the reference gene) when patients (*n* = 7) were compared to controls (*n* = 8). The patient labeled as XLA05 was excluded from this RT-qPCR experiment because of the total consumption of his RNA template.

## Discussion

The definitive diagnosis of XLA is performed by sequencing techniques since in 90–95% of cases, it is possible to identify gene mutations in *BTK* 32.

According to our results, mutations in *BTK* that create a premature stop codon (PTC) can be differentiated due to the reduced level of *BTK* mRNA expression (Fig. 1). These findings are explained by nonsense codons causing a reduction in the mRNA level 33. The nonsense-mediated mRNA decay (NMD) is a surveillance pathway whose main function is to reduce errors in gene expression by eliminating mRNA transcripts that contain PTCs. According to the literature, it is estimated that 30% of known mutations associated with disease are because of mRNAs containing PTCs 34.

At the protein level, flow cytometry assays revealed a downregulation of BTK for all patients (Table1). We also found that mutations in *BTK* lead to a lack of BTK protein or a nonfunctional protein synthesis, evidenced by abnormal subcellular localization of BTK protein in the cell cytoplasm (Fig. 2). In its active state, BTK is found close to the inner surface of the plasma membrane. In fact, BTK phosphorylation is linked to membrane localization 35,36.

Proteins which do not have the required conformation are targets of the cellular mechanism which is designated endoplasmic-reticulum-associated protein degradation (ERAD). This mechanism retrotranslocates misfolded proteins from the ER to the cytosol in order to be degraded via the ubiquitin-proteasome (a protein-degrading complex) 37. Moreover, the increase of unfolded proteins in the ER creates an imbalance between the demand and capacity of the organelle, which is known as ER stress. This triggers a network of signaling events collectively called UPR.

Since ER stress pathways have been implicated in inflammatory diseases 14 but not yet studied in XLA patients, we evaluated by RT-qPCR, the monocyte expression of 12 genes involved in UPR in XLA patients. These genes encompass the three major ER stress branches: ERN1, EIF2AK3, and ATF6, which mediate changes in gene expression that characterize the UPR process 38. In general, we found a hyporegulation scenario for their expressions but *XPB1* had markedly upregulation (Fig. 3). Accordingly, this significant monocyte *XBP1* mRNA overexpression immediately ex vivo could suggest that BTK deficiency may produce ER stress. On the other hand, XBP1 can be expressed independently of the ER-stress response.

Toll-like receptor activation causes XBP1 splicing to increase, XBP1 protein to rise, and pro-inflammatory cytokine transcripts to be generated without initiating ER stress responses. As BTK directly interacts with TLRs and may inhibit TLR-induced cytokine production, as suggested by Marron et al. 39, this might be one important explanation for our observations (Fig. 3), which would indicate that the increase in *XBP1* transcripts in XLA patients is independent of classical ER-stress response. Moreover, downstream ER-stress target gene expressions, such as *HSPA5* and *DDIT3*, were not significantly altered in our samples, which is evidence that the observed *XBP1* overexpression may have occurred in the absence of a classic ER-stress response.

**Figure 4:**
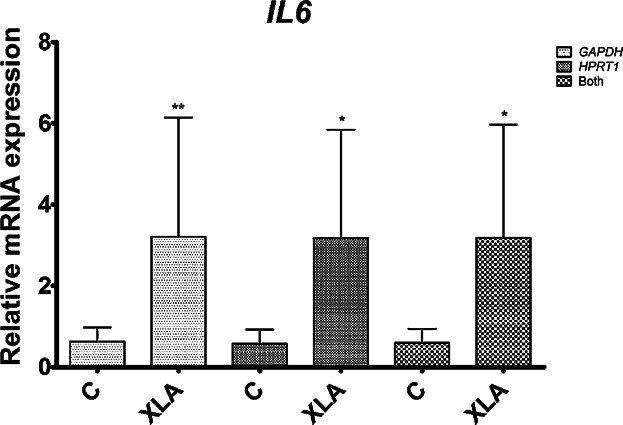
Expression of *IL6* in PBMC from XLA patients and healthy controls. Relative expression of the *IL6* gene in XLA patients (XLA; *n* = 7) and controls (C; *n* = 8). The *y*-axis represents the quantitative data of the relative mRNA expression of the *IL6* gene. Data from RT-qPCRs were generated from three reference genes: *GAPDH*, *HPRT1*, and the combination of *GAPDH* and *HPRT1* (shown as “Both” in the legend). Data are shown as mean and SD. Statistical analysis of the difference between the two groups was performed using the Mann–Whitney test (*P < 0.05; **P < 0.01).

There are no reports in the literature linking BTK and XBP1. Our results prospectively uncover a new mechanism for XBP1 and BTK in the innate immune system. Martinon et al. 25 demonstrated a new function of the IRE1-XBP1 pathway in macrophages that is ER-stress independent. They reported that TLR2 and TLR4 specifically activated XBP1 which amplifies TLR signaling by enhancing cytokine production. Also, the *IL6* overexpression found in our XLA patients (Fig. 4) corroborates the suggestion of BTK repressing TLR-induced cytokine production 39.

Iwakoshi et al. 40 demonstrated that sXBP1 induces IL6 secretion, suggesting that, in addition to its important role in UPR, sXBP1 regulates the expression of this cytokine that is essential for plasma cell differentiation and myeloma cell growth. The authors raised an interesting hypothesis in which XBP1 has unidentified functions and target genes that are unrelated to UPR, reinforced by the fact that its role in driving the transcription of at least a subset of ER chaperone genes is redundant 40. Interleukin 6 might be one such target. Our results also support their hypothesis of the existence of a positive feedback loop between IL6 and XBP1 (that would ensure high quantities of both proteins during plasma cell differentiation). Since plasma cell differentiation is defective in XLA patients, the overexpression of both these genes could represent a physiological attempt to correct this failure. In addition, as IL6 is a potent pro-inflammatory cytokine and XBP1 a multifunctional transcription factor, their involvement in XLA physiopathology is conceivable and further studies should be undertaken.

Before adequate immune globulin treatment, several inflammatory conditions were described as being associated with XLA. Recent data from 128 unique XLA patient responses indicated that a considerable number of XLA patients have symptoms that are consistent with a diagnosis of arthritis, inflammatory bowel disease, or other inflammatory conditions 41. The suggested positive feedback loop between IL6 and XBP1 should be further investigated in these patients.

In response to LPS, PBMC from XLA patients produced significantly higher amounts of pro-inflammatory cytokines and IL10 compared to controls, and this production was influenced neither by the severity of the mutation nor the affected domain 42. The authors of this study also demonstrated a predominantly inflammatory response in XLA patients after LPS stimulation and suggested a deregulation of TLR signaling in the absence of BTK. Environmental factors were suggested to be involved in this response 42.

Schmidt et al. 43 demonstrated that Btk is activated by Tlr4 in primary macrophages and is required for normal Tlr-induced Il10 production in multiple macrophage populations. The authors suggest that the decreased Il10 production may be responsible for increased Il6. BTK also plays a critical role in initiating TLR3 signaling. In the absence of BTK, TLR3-induced phosphoinositide 3-kinase (PI3K), AKT and MAPK signaling, activation of NFκB as well as interferon regulatory factor 3 (IRF3), and AP-1 transcription factors were all defective. It was demonstrated that BTK directly phosphorylates TLR3 and, in particular, the critical Tyr759 residue. Loss of BTK also compromises the formation of the downstream TRIF/receptor-interacting protein 1 (RIP1)/TBK1 complex 44. Since some XLA patients develop enterovirus encephalitis 45, the participation of a defective TLR3 signaling should be considered as a potential molecular pathomecanism in this condition.

Recently, Savic et al. 46 reported the first link between TLR-dependent XBP1 activation and human inflammatory disease. They showed that TLR-dependent XBP1 activation is operative in the synovial fibroblasts (SF) of active rheumatoid arthritis (RA) patients. They found that the active (spliced) form of (s)XBP1 was significantly upregulated in the active RA group compared to healthy controls and patients in remission. Interestingly and paradoxically, the expression of nine other ER stress response genes was reduced in active RA compared to patients in remission, suggestive of a UPR-independent process 46. Our results clearly suggest a similar scenario for XLA patients, who experience autoimmune disease and BTK deficient monocytes are known to be pro-inflammatory.

Moreover, the Savic et al. 46 study demonstrated that sXBP1 was induced in SF by TLR2 and TLR4 stimulation, resulting in sXBP1-dependent IL6 and TNF production. Further studies are necessary to shed light on the mechanism by which sXBP1 controls IL6 expression. Spliced XBP1 protein can confer on B cells the ability to produce IL6; however, a comparison of wildtype and *XBP1*^−/−^ B cells stimulated with LPS in vitro showed no significant difference in IL6 production. Thus, XBP1 is not essential for expression of *IL6* 40 and it is well known that several transcription factors, such as CCAAT/enhancer binding protein (C/EBP), beta (CEBPB), and NFκB among others, regulate *IL6* expression 47.

We also found a downregulation of *HSP90B1*. The protein encoded by this gene participates in the activation of chaperones by the ATF6 and the immune response antigen presentation by MHC class I pathways. There is evidence of its involvement in some autoimmune and inflammatory diseases 48. More experiments could explain the role of the monocyte *HSP90B1* downregulation found in our XLA patients; however, this result corroborates with the ruling out of the participation of the ATF6 ER stress pathway in these patients. Complementarily, monocyte *ATF6* expression was not significantly different when XLA patients and controls were compared (Fig. 3). Indeed, as stop codon *BTK* mutations lead to an absence of the BTK protein, as seen in Figure 2, an UPR activation in the affected cells is not expected. On the other hand, we do not discard an association of ER stress pathways when the BTK protein is misfolded due to missense mutations. A larger or multicentric study including more patients could elucidate this question.

## Conclusions

Up to now, the literature has implied that BTK is critical for B cell development and differentiation, but is also involved in the regulation of other cell types, such as monocytes/macrophages 49, neutrophils 50, and NK cells 51. BTK has also been implicated in TLR signaling and interacts with TLR4, 6, 8, and 9 and also with MYD88, toll-interleukin 1 receptor (TIR) domain containing adaptor protein (TIRAP) (also known as MyD88-adaptor-like protein [Mal]) and interleukin-1 receptor-associated kinase 1 (IRAK1) 52, indicating unexplored roles for this protein in innate and adaptive immunity.

We demonstrated that *BTK* mRNA is markedly downregulated in XLA patients with stop codon mutations and that missense mutations do not affect *BTK* expression. Moreover, this is the first study relating XBP1 with *BTK* mutations and XLA. Monocytes from XLA patients overexpress *XBP1*, a critical transcriptional factor for ER stress and plasma cell differentiation. Our data suggest that defective BTK might affect XBP1 expression in monocytes. As a multifunctional transcriptional factor, our finding on XBP1 upregulation in XLA patients opens several possible avenues of research that will help us to understand the complex pathophysiology in XLA.
